# Clinical, radiographic characteristics and immunomodulating changes in neuromyelitis optica with extensive brain lesions

**DOI:** 10.1186/1471-2377-13-72

**Published:** 2013-07-03

**Authors:** Chen Cheng, Ying Jiang, Xiaohong Chen, Yongqiang Dai, Zhuang Kang, Zhengqi Lu, Fuhua Peng, Xueqiang Hu

**Affiliations:** 1 Multiple sclerosis center, Department of Neurology, the Third Affiliated Hospital, Sun Yat-sen University, 600 Tianhe Road, Guangzhou, Guangdong 510630, China; 2 Department of Radiology, the Third Affiliated Hospital, Sun Yat-sen University, 600 Tianhe Road, Guangzhou, Guangdong 510630, China

**Keywords:** Aquaporin-4, Complement, C-reactive Protein, Erythrocyte sedimentation rate, Extensive brain lesions, Magnetic resonance imaging, Neuromyelitis optica

## Abstract

**Background:**

Neuromyelitis optica (NMO) shows various brain magnetic resonance imaging (MRI) abnormalities with recurrent central nervous system (CNS) attacks, although predominantly affecting the spinal cord and optic nerve. However, NMO with extensive involvement of the brain has infrequently been studied. We investigated the clinical, radiographic features and immunomodulating changes of NMO patients with extensive brain lesions (EBLs) in China.

**Methods:**

NMO patients (including 16 NMO patients with EBLs and 53 NMO patients without EBLs) hospitalized during January 2006 and February 2010 were recruited and analyzed retrospectively. Data of clinical characteristics, magnetic resonance imaging (MRI) features, laboratory abnormalities, treatment details and outcomes were analyzed. All the patients received the follow-up visits for two years.

**Results:**

EBLs in NMO were classified into four categories according to their respective MRI characteristics: 1) Tumefactive-like lesions (n=4, 25%); 2) Acute disseminated encephalomyelitis (ADEM)-like lesions (n=6, 37.5%); 3) Multiple sclerosis (MS)-like lesions (n=5, 31.25%); 4) Posterior reversible encephalopathy syndrome (PRES)-like lesions (n=1, 6.25%). NMO patients with EBLs had higher rates of encephalopathy symptoms (37.5% vs. 5.6%, p = 0.004), homonymous hemianopia (18.8% vs. 0%, p = 0.011) and AQP4 seropositivity (100% vs. 69.8%, p = 0.008) than NMO patients without EBLs (NEBLs). Immunomodulating changes (including the levels of C3, C4, ESR and CRP) were significantly higher in patients with EBLs than those without EBLs. The relapse times in EBLs during the follow-up period were more frequent than those happened in NEBLs (1.88 ± 0.30 vs. 1.23 ± 0.14, p = 0.04). The EDSS scores in EBLs patients were also much higher than those in NEBLs throughout all the whole visits of follow-up.

**Conclusions:**

The presence of EBLs in NMO may indicate a higher diseases activity and portend a worse prognosis. CRP is a useful marker in monitoring diseases activity. Systemic inflammation may be crucial to the formation of EBLs in NMO.

## Background

Neuromyelitis optica (NMO) is an autoimmune inflammatory and demyelinating disorder of the central nervous system (CNS), predominantly affecting the optic nerves and spinal cord. Evidence from several sources (clinical, imaging, immunologic and immunopathologic data) demonstrate that NMO is certainly a distinct disease with a different pathogenesis from multiple sclerosis (MS) [1]. The concept of NMO has been changed by the detection of an antibody against the water channel protein, anti-aquaporin-4 (AQP4) antibody [2,3], which reveals that the involvement of brain is not uncommon in NMO [4,5]. Moreover, several kinds of brain lesions detected by magnetic resonance imaging (MRI) have been considered as characteristics of NMO [4,6]. Among them, atypical brain lesions, such as large confluent lesions (≥3 cm) and diencephalic lesions, have been reported to show vasogenic edema without enhancement in AQP4-seropositive patients [4,7,8]. However, NMO with extensive involvement of the brain has only infrequently been studied on a large-sample basis, especially in the differences between the NMO patients with extensive brain lesions (EBLs) and those who without EBLs.

The distributions of atypical brain lesions on MRI reflect the distributions of high expression of AQP4 [6,9]. And histopathological analysis of NMO show perivascular IgM and IgG deposition with complement activation and loss of immunoreactivity to AQP4 [10-12]. These findings suggest that humoral immunity and anti-AQP4 antibody play important roles in the pathogenesis of NMO. Anti-AQP4 antibodies mainly belong to the IgG1 subclass and complement-mediated injury is postulated [13]. In other autoimmune diseases, such as systemic lupus erythematosus (SLE) and Sjögren’s syndrome (SS), the serum level of complement decrease at relapse because of consumption. And this can help to predict the prognosis of these diseases [14-17]. C-reactive protein (CRP) is a component of the innate immune system, which reflects systemic low-grade inflammatory activity [18]. Erythrocyte sedimentation rate (ESR) is a less-specific marker of systemic inflammation, which is known to elevate in many acute or chronic diseases characterized by tissue necrosis and inflammation [18]. CRP and ESR are considered as two general markers of inflammatory activity [19,20]. Serum level of CRP was reported to be moderately increased in patients with MS and correlate with disease activity [21], while serum level of ESR was slightly higher in female MS patients than male patients [22]. However, there have been no reports about the serum complement, CRP and ESR levels in NMO patients with EBLs.

In the present study, we for the first time investigated the clinical features and MRI characteristics in NMO patients with EBLs and compared the serum levels of CRP, ESR and complement (including C3, C4 and CH50) in NMO patients with EBLs with those without EBLs.

## Methods

### Patients

We retrospectively reviewed the medical records of 16 patients with EBLs (all of the patients were AQP4 seropositive) and 53 patients without EBLs (including 37 AQP4 seropositive patients and 16 AQP4 seronegative patients) from January 2006 to February 2010 admitted to the MS center of the Third affiliated hospital of Sun Yat-sen University in Guangzhou, China. All the cases were diagnosed in accordance with the Wingerchuk 2006 revised criteria [23] and followed up for 2 years. None of the patients had anemia, hypoalbuminemia or any systemic complications (such as an upper respiratory tract infection, pneumonia or lower urinary tract infection). Data collection was approved by the institution’s ethics committee. Clinical features and outcomes were recorded in details, including sex, age at onset, duration, relapse times, annualized recurrence rate, clinical manifestations of brain involvement and functional outcomes assessed according to the Expanded Disability Status Scale (EDSS) [24]. The assessment of EDSS in all the patients had been followed up for 2 years since the emergence of EBLs or clinical symptoms relating to NMO.

### Magnetic resonance imaging (MRI) scanning

A 1.5-T magnetic resonance imager (General Electric, Milwaukee, WI, USA) was used to perform the brain and spinal cord MRIs. Conventional MRI protocols were applied to all the patients, such as T1-weighted images (T1W) with and without gadolinium enhancement (GDE), T2-weighted images (T2W) and fluid attenuated inversion recovery (FLAIR). Nonconventional MRI protocols [including diffusion-weighted imaging (DWI) and apparent diffusion coefficient (ADC) maps] were applied to nine NMO patients with EBLs.

All the patients were performed with brain and spinal MRIs. All the 16 EBLs were considered as acute lesions. “Acute relapse” was defined as a permanent functional deficit exited definitely within the first eight weeks [25]. There were ten (62.5%) NMO patients with EBLs accompanied with symptoms of brain involvement within seven days. Other six patients (37.5%) with EBLs did not manifest the corresponding brain symptoms. However, the lesions didn’t emerge in the pre-scanning of brain MRI less than eight weeks ago. Brain MRIs of nine patients with EBLs had been followed up after the emergence of EBLs.

Extensive brain lesions, such as a large confluent cerebral hemisphere lesions or confluent diencephalic lesions (involving the thalamus and hypothalamus), which is 30 mm or more in diameter on FLAIR or T2W, are based on previous reports [4]. The EBLs were classified into four categories according to their different MRI characteristics: 1) Tumefactive-like lesions, which are considered as solitary lesions mimicking neoplasms, greater than 30 mm in diameter with little mass effect or edema [26]; 2) Acute disseminating encephalomyelitis (ADEM)-like lesions, which are generally thought as extensive bilateral white matter lesions lacking in enhancement following gadolinium injection [27]; 3) MS-like lesions, which are defined as asymmetric and multifocal lesions with a predilection for the periventricular and subcortical white matter [4]; 4) Posterior reversible encephalopathy syndrome (PRES)-like lesions, which are considered as T2W hyperintense signal abnormalities primarily located at parieto-occipital/cerebellar regions and would completely resolve in four weeks [28].

### Laboratory assessment of complement, ESR and CRP

The serum complement, ESR and CRP of all the patients were measured at the time of clinical relapse (within 14 days of acute relapse). None of systemic complications, such as upper respiratory tract infections, pneumonia or lower urinary tract infections, were found in patients when blood samples were collected. C3 level and C4 level were determined by turbid metric immunoassay, and CH50 activity was determined by liposome immunoassay. ESR value was determined by applying routine methods. CRP level was measured by latex photometric immunoassay. Normal ranges were 0.8-1.6 g/L for C3, 0.1-0.4 g/L for C4, 23–46 U/ml for CH50, 0-20 mm/H for ESR and 0–6 mg/L for CRP.

### AQP4 and oligoclonal bands testing

Serums from all subjects were tested for anti-AQP4 antibody by a commercial sampling kit (Euroimmun, Germany) according to the manufacturer’s instructions. The CSF oligoclonal bands (OCB) detection method used in our laboratory is an isoelectric focusing technique combined with the avidin-biotinperoxidase complex method.

### Statistical analysis

All quantitative data in this study were presented as (i) mean ± standard deviation (SD), or (ii) median (min to max). The chi-square test and Fisher’s exact test were used to compare categorical values. For comparison of continuous values, we used the Mann–Whitney U-test for non-normally distributed variables and two independent-Samples T-test for normally distributed variables. The correlation between EDSS and CRP /ESR level was analyzed by Spearman’s rank correlation test. Values of P < 0.05 were considered statistically significant. Statistical analysis was performed by using SPSS 16.0 (SPSS Inc, Chicago, IL, USA) for Windows.

## Results

### Main characteristics of the study population

All the subjects were classified into three groups: 1. EBLs (16 NMO patients with EBLs); 2. NEBLs (53 NMO patients without EBLs); 3. NEBLs* (37 AQP4-seropositive NMO patients without EBLs, all of whom are included in NEBLs). The clinical features of these NMO patients were summarized in Table 1.

**Table 1:** Demographic and clinical features of EBLs, NEBLs and NEBLs*

**Variable**	**EBLs (n=16)**	**NEBLs (n=53)**	NEBLs* (n=37)	**P 1**	**P2**
Age at symptom onset (years)	33.0±1.55	32.8±1.42	35.3±1.42	NS	NS
Sex(Male/Female)	2/14	7/46	4/33	NS	NS
Annualized relapse rate	2.11 ± 2.0	1.17 ± 0.6	1.10 ± 1.15	NS	NS
Relapse times	5(3–18)	5(2–16)	5(2–16)	NS	NS
Duration (months)	82.4±5.2	83.5±6.0	99.3±6.34	NS	NS
Brain symptom(%)	10(62.5%)	27(50.1%)	20(54.1%)	NS	NS
encephalopathy symptom	6(37.5%)	3(5.6%)	3(8.1%)	0.004	0.016
brain-stem symptom	5(31.2%)	20(37.7%)	13(35.1%)	NS	NS
cerebellum involvement	2(12.5%)	4(7.5%)	3(8.1%)	NS	NS
homonymous hemianopia	3(18.8%)	0(0)	0(0)	0.011	0.024
EDSS*	2.49 ± 1.03	1.98± 0.97	2.00 ± 1.04	NS	NS
EDSS**	7.67 ± 1.48	5.32 ± 1.82	5.32 ± 1.82	0.000	0.000
Serum Analysis					
CRP (mg/l)	12.32 ± 3.05	2.65 ± 0.61	3.01 ± 1.01	0.000	0.000
ESR (mm/H)	27.67 ± 16.49	15.83 ± 11.28	15.24 ± 12.24	0.005	0.004
C3 (g/l)	1.42 ± 0.50	1.15 ± 0.27	1.14 ± 0.30	0.049	0.043
C4 (g/l)	0.29 ± 0.16	0.22 ± 0.09	0.22 ± 0.07	0.046	NS
CH50 (U/ml)	50.73 ± 10.62	45.96 ± 11.32	44.43 ± 11.52	NS	NS
AQP4 seropositivity	16(100%)	37(69.8%)	37(100%)	0.008	
CSF Analysis					
CSF-TP (mg/ml)	0.41 ± 0.25	0.29 ± 0.17	0.31 ± 0.18	NS	NS
CSF-WBC (10)	6.0(0–58)	5(0–98)	5(0–46)	NS	NS
CSF-OCB	1(6.25%)	3(5.66%)	2(5.40%)	NS	NS

*EBLs* neuromyelitis optica with extensive brain lesions; NEBLs=neuromyelitis optica without extensive brain lesions; NEBLs*=neuromyelitis optica without extensive brain lesions and with a positive Anti-AQP4; *EDSS* Expanded Disability Status Scale; *NS* no significant; EDSS* 6 months before EBLs presentation or clinical onset; EDSS** at EBLs presentation or clinical onset; *AQP4* aquaporin-4; *CSF* cerebrospinal fluid, CSF taken during EBLs presentation or clinical onset; P1=EBLs vs NEBLs; P2=EBLs vs NEBLs*.

Among the 16 patients with EBLs, ten of them (62.5%) had clinical manifestations of brain involvement (Table 1), which could be classified into four types: 1) encephalopathy symptoms (n=6), including narcolepsy, coma, seizure and headache; 2) brainstem symptoms (n=5), including intractable hiccup, nausea and vomiting and bulbar dysfunction; 3) homonymous hemianopia (n=3); 4) cerebellum involvement (n=2), mainly manifested as ataxia. Encephalopathy symptoms and homonymous hemianopia were more common in patients with EBLs (Table 1).

**Figure 1:**
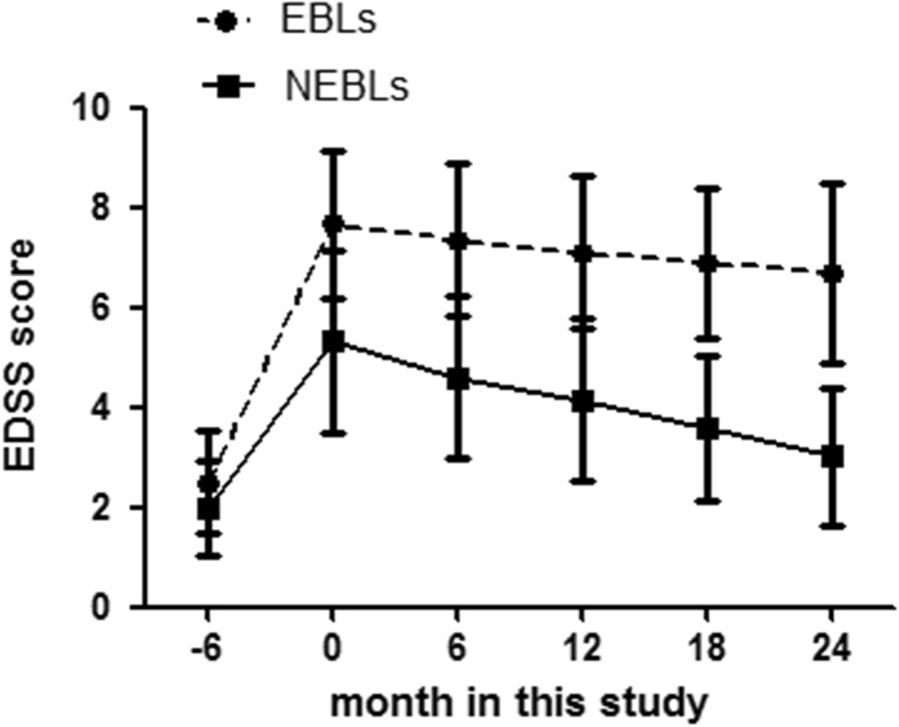
**The mean±SD EDSS values during a 2 years of follow-up after EBLs presentation or clinical onset in EBLs and NEBLs.** EBLs, neuromyelitis optica with extensive brain lesions; NEBLs, neuromyelitis optica without extensive brain lesions.

Table 1 presented the EDSS scores before EBLs attack and the EDSS scores assessed at the emergence of EBLs or clinical onset in EBLs, NEBLs and NEBLs* respectively. The EDSS values of EBLs and NEBLs during 2 years of follow-up were shown in Figure 1 and Table 2.

**Table 2:** Treatment and prognosis in EBLs, NEBLs and NEBLs*

**Variable**	**EBLs (n=16)**	**NEBLs (n=53)**	NEBLs* (n=37)	**P1**	**P2**
Therapy in relapse					
High-dose corticosteroids pulses	16(100%)	53(100%)	37(100%)	NS	NS
Combined with IVIG	6(37.5%)	17(32.0%)	11(29.7%)	NS	NS
Plasmapheresis	1(6.25%)	1(1.89%)	1(2.70%)	NS	NS
Therapy in remission					
Small-dose prednisone(8–20 mg/d)	16(100%)	53(100%)	37(100%)	NS	NS
Combined with Azathioprine(50 mg/d)	6(37.5%)	24(47.2%)	19(51.4%)	NS	NS
Prognosis					
NO. of relapse cases	12(75%)	39(73.5%)	31(79.4%)	NS	NS
Relapse times after emergence of EBLs or symptoms relating to NMO	1.88±0.30	1.23±0.14	1.36±0.16	0.04	NS
NO. of spinal cord attack	1.31±0.25	0.71±0.12	0.81 ± 0.14	0.021	NS
NO. of optic nerves attack	0.56±0.18	0.26±0.07	0.21 ± 0.08	NS	0.039
NO. of Brain attack	0.43±0.16	0.37±0.09	0.40±0.11	NS	NS
EDSS score					
EDSS in 6 month	7.35± 1.48	4.6±1.63	4.62±1.64	0.001	0.001
EDSS in 12 month	7.1±1.53	4.15±1.64	4.16±1.59	0	0
EDSS in 18 month	6.88±1.49	3.58 ± 1.47	3.58 ± 1.47	0	0
EDSS in 24 month	6.70 ± 1.80	3.01 ± 1.36	3.03 ± 1.49	0	0
NO. of patients decreased EDSS ≥ 3	3(18.75%)	18(33.96.16%)	13(35.13%)	NS	NS
(from onset to the last visit)					

*EBLs* neuromyelitis optica with extensive brain lesions; NEBLs=neuromyelitis optica without extensive brain lesions; NEBLs*=neuromyelitis optica without extensive brain lesions and with a positive Anti-AQP4; *EDSS* Expanded Disability Status Scale; P1=EBLs vs NEBLs; P2=EBLs vs NEBLs*.

### The MRI features of EBLs in NMO

**Figure 2:**
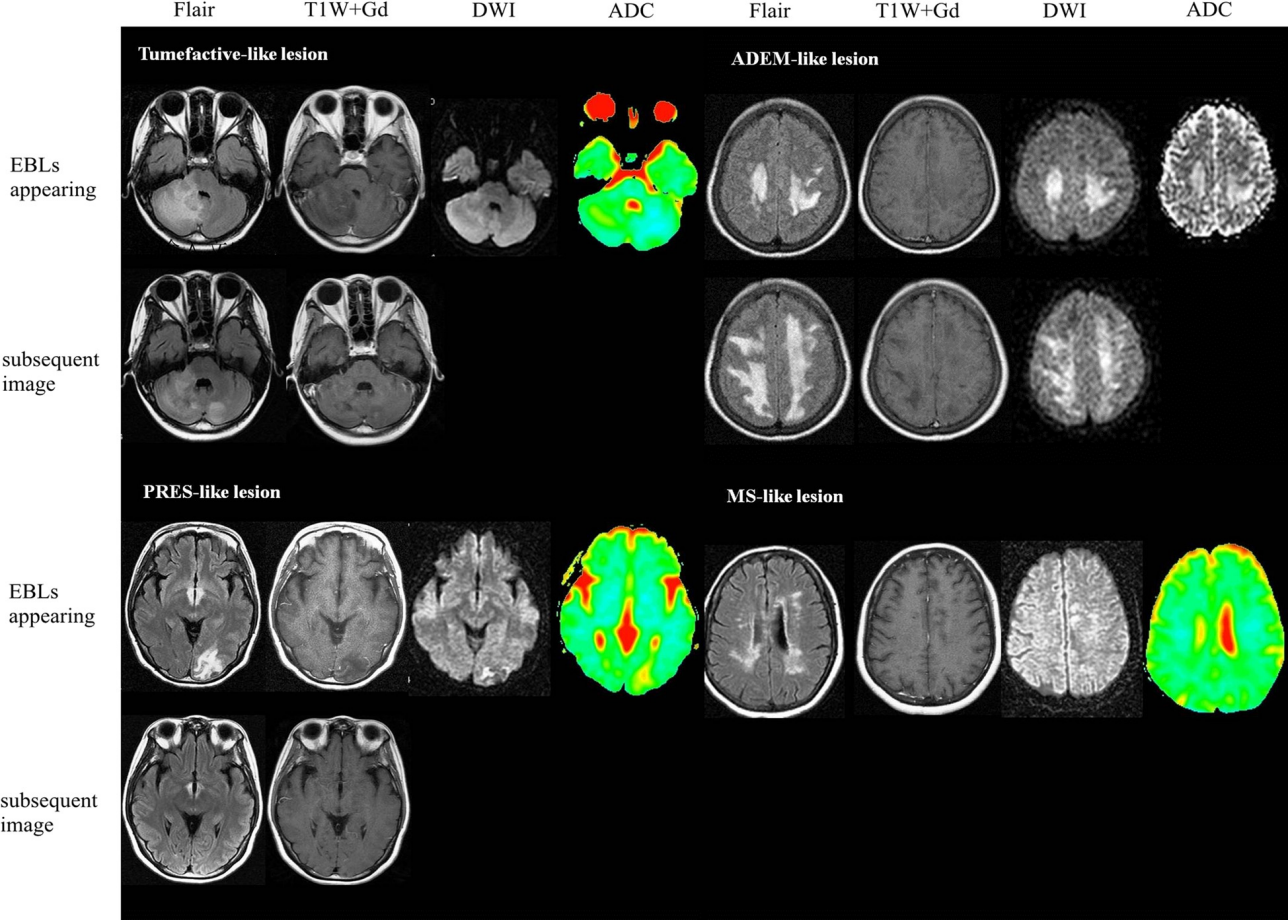
**Typical four typies images of EBLs are displayed according to the following MRI sequences, as available: fluid-attenuated inversion recovery (FLAIR), T1W+Gd, diffusion-weighted imaging (DWI), apparent diffusion coefficient.** The top rows represent neuroimaging at time of EBLs appeared, and the bottom rows represent post-EBLs repeated neuroimaging. Tumefactive-like lesion: No enhanced tumefactive-like lesions in cerebellar hemisphere shrink in repeating imagings of two months later. ADEM-like lesion: No enhanced extensive bilateral white matter brain lesions are markedly expanded in the subsequent MRI of three months later. PRES-like lesion: No enhanced lesions in left occipital lobe completely resolve in less than four weeks. MS-like lesion: No enhanced multifocal lesions locate in the periventricular and subcortical white matter. The MR images of follow-up are not supplied. The DWI, ADC images and MR images of follow-up are not supplied. All the DWI and ADC images reveal changes consistent with vasogenic edema.

According to MRI findings, the EBLs were divided into four categories (Table 3): 1) Tumefactive-like lesions (n=4, 25%); 2) ADEM-like lesions (n=6, 37.5%); 3) MS-like lesions (n=5, 31.25%); 4) PRES-like lesions (n=1, 6.25%). The MRI features of EBLs in NMO were shown in Table 3. Images of four typical EBLs were displayed in Figure 2.

**Table 3:** MRI features of NMO patients with EBLs

**Radiologic features of the EBLs**	**n=16%**
Acute lesions	16(100%)
Tumefactive-like lesions	4(25%)
ADEM-like lesions	6(37.5%)
MS-like lesions	5(31.25%)
PRES-like lesions	1(6.25%)
Margins	
Well defined	9(56.25%)
Diffuse	7(43.75%)
Mass effect	
None	13(81.25%)
Moderate	2(12.5%)
Marked	1(6.25%)
Singnal of the EBLs	
T1W hypointense	16(100%)
T2W hyperintense	16(100%)
T2FLAIR hyperintense	16(100%)
Enhanced pattern	
Open ring	1(6.25%)
None	15(93.75%)
Nonconventional Neuroimaging of EBLs (n=9)	
High intense signal in DWI	9(100%)
ADC value increased	9(100%)

One EBLs appeared with GDE, which manifested as open-ring like pattern and was observed in one patient (25%) with tumefacive-like lesions. Contrast enhancement was absent in ADEM-like lesions, MS-like lesion and PRES-like lesions.

Brain MRIs of nine patients with EBLs had been followed up when EBLs emerged. On the subsequent MRIs, the size of EBLs were markedly reduced or resolved in four patients (44.4%, including two Tumefactive-like lesions, one PRES-like lesions and one MS-like lesions), expanded in two patients (22.2%, two ADEM-like lesions), and unchanged obviously in other three patients (33.3%, including two ADEM-like lesions and one MS-like lesions).

### Serum complement levels

**Figure 3:**
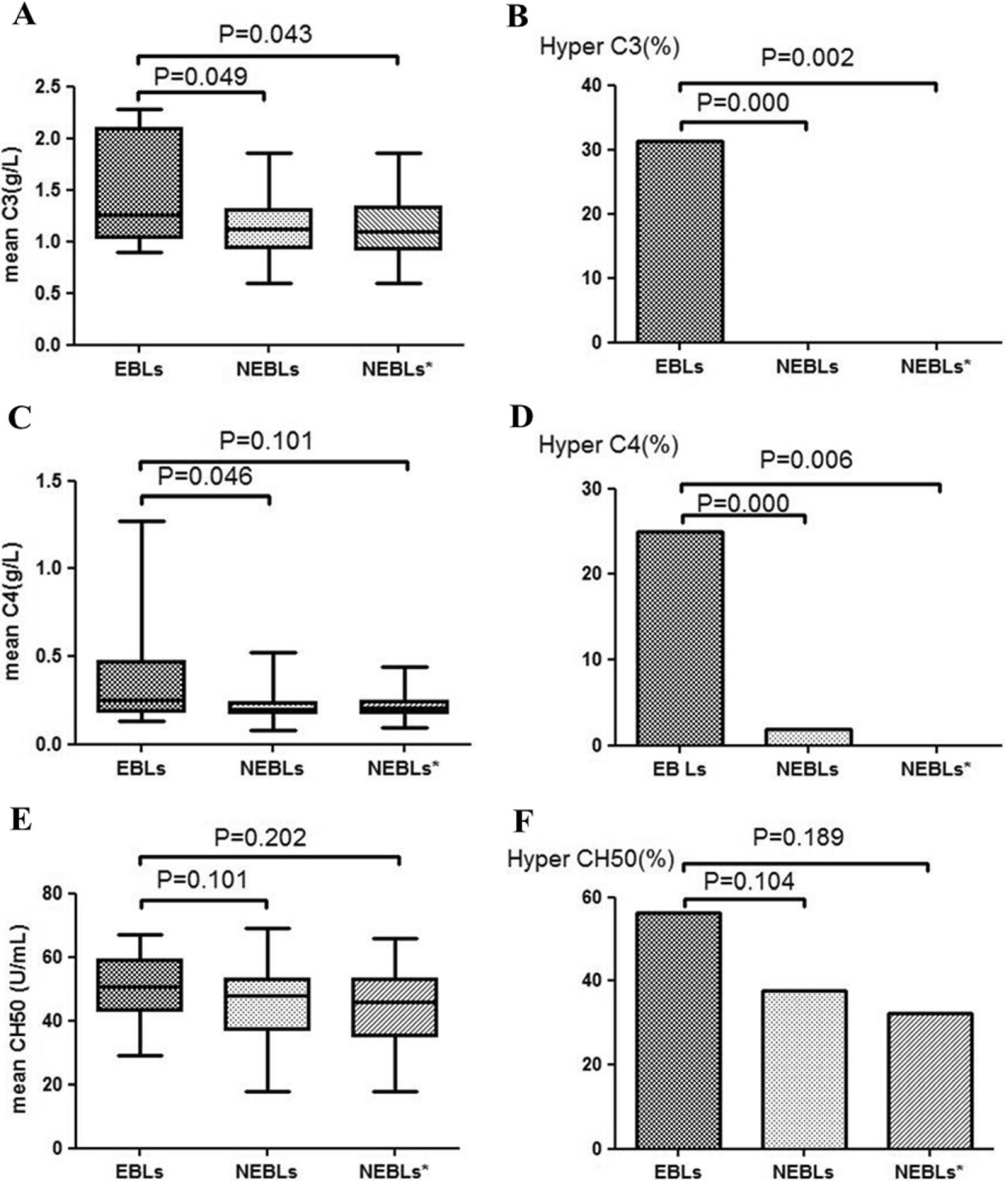
(A, C, E) C3, C4 and CH50 values and (B, D, F) the frequencies of patients with high C3, C4 and CH50 values among EBLs, NEBLs and NEBLs*. EBLs; neuromyelitis optica with extensive brain lesions; NEBLs: neuromyelitis optica without extensive brain lesions; NEBLs*:AQP4-seropositive neuromyelitis optica without extensive brain lesions.

The data about C3, C4 and CH50 values and the frequencies of patients with high C3 (C3 >2.0 g/L), C4 (C4 >0.5 g/L) and CH50 (CH50 >50 U/ml) values among EBLs, NEBLs and NEBLs* were shown in Table 1 and Figure 3. There were no significant correlation between serum C3, C4, CH50 levels and EDSS scores in NMO with EBLs and without EBLs. The data about C3, C4 and CH50 values between AQP4- seropositive NEBLs (n=37) and AQP4-seronegative NEBLs (n=16) were shown in Table 4. There were no significant differences in serum C3, C4 and CH50 levels between AQP4-seropositive NEBLs and AQP4-seronegative NEBLs. There were also no significant differences between AQP4-seropositive NMO (n=53) and AQP4-seronegative NMO (n=16) in serum levels of C3, C4 and CH50 (Table 4).

**Table 4:** Comparison of Serum Analysis between AQP4 (−) NEBLs patients and AQP4 (+) NEBLs patients

**Variable**	**AQP4(−) NEBLs patients (n=16)**	**AQP4(+) NEBLs patients (n=37)**	**P values**
Serum analysis			
CRP (mg/l)	1.81 ± 0.65	3.01 ± 1.01	0.371
ESR (mm/H)	17.19 ± 2.22	15.24 ± 12.24	0.57
C3 (g/l)	1.16 ± 0.20	1.14 ± 0.30	0.838
C4 (g/l)	0.29 ± 0.16	0.22 ± 0.07	0.772
CH50 (U/ml)	49.50 ± 10.30	44.43 ± 11.52	0.136

*AQP4* (−) anti-aquaporin-4 antibody negative; AQP4 (+) = anti-aquaporin-4 antibody positive EBLs = neuromyelitis optica with extensive brain lesions; NEBLs = neuromyelitis optica without extensive brain lesions; *AQP4* aquaporin-4.

### Serum CRP, ESR levels

**Figure 4:**
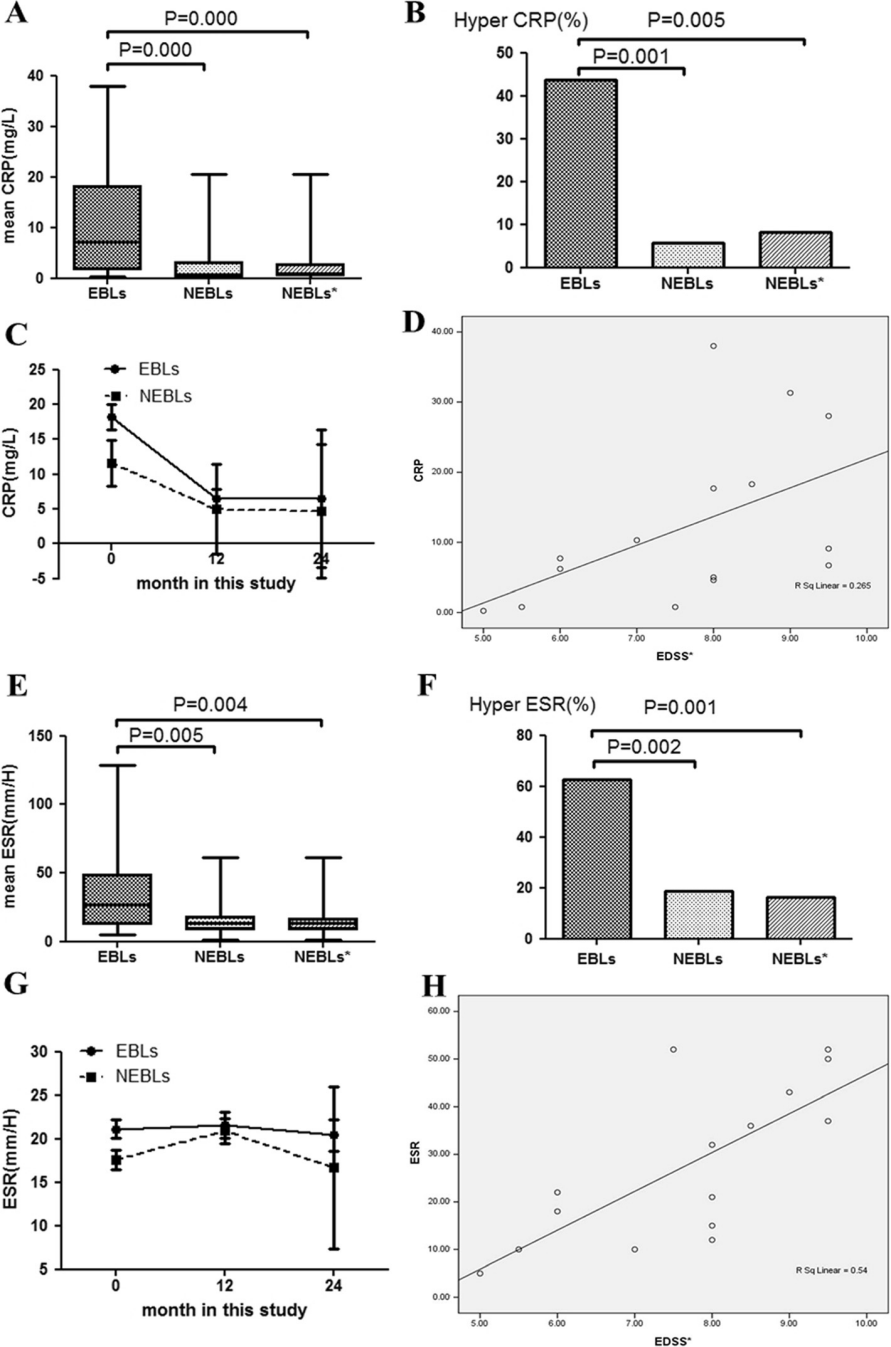
CRP (A) and ESR (E) values and the frequencies of patients with high CRP (B) and ESR (F) values among EBLs, NEBLs and NEBLs*. Serum CRP **(C)** and ESR **(G)** values during the 2 years of follow-up in EBLs and NEBLs. Correlation between serum CRP **(D)** and ESR **(H)** values and EDSS scores of EBLs. EBLs: neuromyelitis optica with extensive brain lesions; NEBLs: neuromyelitis optica without extensive brain lesions; NEBLs*: AQP4 seropositive neuromyelitis optica without extensive brain lesions.

The data of CRP and ESR values and the frequencies of patients with high CRP (CRP >10 mg/L) and ESR (ESR > 20 mm/H) values among EBLs, NEBLs and NEBLs* were presented in Table 1 and Figure 4. CRP and ESR values during the 2 year follow-up of EBLs and NEBLs were presented in Figure 4. Significantly positive correlations were identified in NMO patients with EBLs between the mean EDSS scores and serum values of each of CRP (r = 0.529, p = 0.02) (Figure 4) and ESR (r = 0.725, p = 0.002) (Figure 4). However, such correlations were not detected in NEBLs and NEBLs*. The data about CRP and ESR values in AQP4-seropositive NEBLs (n=37) and AQP4-seronegative NEBLs (n=16) were shown in Table 4. There were no significant differences in serum CRP and ESR levels between AQP4-seropositve NEBLs and AQP4-seronegative NEBLs. There were also no significant differences between AQP4-seropositive NMO (n=53) and AQP4-seronegative NMO (n=16) in serum CRP, ESR levels (Table 4).

### Treatment and outcome

The therapies in patients with EBLs, NEBLs and NEBLs* were shown in Table 2. The therapies of NMO patients with EBLs and those without EBLs were almost the same in our center. All of the patients (100%) received high-dose corticosteroids pulses [(methylprednisolone 1 g, IV /d for 5d) for 2–3 courses; each treatment interval was three days] during the relapse period. In the EBLs group, the treatments for six patients were combined with IVIG (0.4 g/kg/d × 3d, used during each treatment interval). One patient was treated with plasmapheresis (2.0 L/times, every other day and continuous 3 times). While in the NEBLs group, the treatments for 17 patients were combined with IVIG (0.4 g/kg/d × 3d, used during each treatment interval). One patient was treated with plasmapheresis. In remission period, all the patients received small doses of prednisone (8 to 20 mg / d, oral). The treatments for six patients with EBLs and 24 patients without EBLs were combined with Azathioprine (50 mg/d).

The data about prognosis of EBLs, NEBLs and NEBLs* patients were shown in Table 2. In the 2-years visit of follow-up, the number of relapse cases was 12 in EBLs, 39 in NEBLs and 31 in NEBLs*. The relapse times were much higher in EBLs than those in NEBLs (1.88 ± 0.30 vs. 1.23 ± 0.14, p = 0.04). Spinal cord attacks happened more frequently in EBLs than those in NEBLs (1.31 ± 0.25 vs. 0.71 ± 0.12, p = 0.021). The frequencies of optic nerves attacks were also much higher in EBLs than those in NEBLs* (0.26 ± 0.07 vs. 0.21 ± 0.08, p = 0.039). However, there was no significant difference in the numbers of brain attacks. The numbers of patients improved significantly (be defined as decreased EDSS ≥ 3 from onset to the last visit) were three in EBLs, 18 in NEBLs and 13 in NEBLs* respectively.

## Discussion

### The clinical features of NMO with EBLs

The clinical symptoms of brain involvement in NMO with EBLs were categorized into four types: 1) encephalopathy symptoms; 2) brain-stem symptoms; 3) homonymous hemianopia; 4) symptoms of cerebellum. The encephalopathy symptoms rarely occurred in NMO. However, six (37.5%) of these 16 patients with EBLs had encephalopathy symptoms, which was much higher than that happened in NMO without EBLs. According to the MRI findings, when the lesions were involved in the functional areas (such as the cortex, brainstem, cerebellum, etc.), the corresponding symptoms happened.

All the NMO patients with EBLs (100%) had seropositivity of anti-AQP4 antibody, which was much higher than that in NMO patients without EBLs (69.8%, p = 0.008). Takahashi et al. reported that serum anti-AQP4 antibody titers were higher in young adult patients (< 45 years old) with extensive or large cerebral lesions on MRI [29]. EDSS scores before EBLs attack were almost the same among these three groups. However, the EDSS scores at the clinical onset were higher in patients with EBLs than those without EBLs. And during the visits of follow-up, the EDSS scores were also higher in patients with EBLs than those without EBLs. These results suggest that the presence of EBLs in NMO may indicate a higher disease activity and portend a worse prognosis than NMO without EBLs.

### The MRI characteristics of NMO with EBLs

In our study, the EBLs were classified into four types according to their MRI characteristics: 1) Tumefactive-like lesions; 2) ADEM-like lesions; 3) MS-like lesions; 4) PRES-like lesions. Contrast-enhancement was absent or faint in three and open-ring in one (25%) among the tumefactive-like lesions (n=4). Open-ring enhancement was considered as a characteristic manifestation of demyelinating lesions [30]. Confluent white matter lesions in six patients were similar to ADEM. Typical ADEM without concurrent NMO were usually observed acute monophasic perivenous non-confluent demyelinating lesions [31]. While ADEM-like lesions in our study showed diffuse white matter lesions with a chronic expansion, which was different from the typical ADEM. The ADEM-like lesions revealed no enhancement in our study, which were also unusual in typical ADEM [32]. The ADEM lesions often had GDE on brain MRI. However, NMO-associated ADEM lesions may lack GDE [27,31]. Brain lesions in NMO have been reported to lack GDE because of the integrity of blood–brain barrier [27].

The presence of diffuse subcortical edema preferentially affecting the parieto-occipital regions in one patient indicated PRES-like radiological features. Previous study has reported PRES occurred in patients with NMOSD [28]. Blood pressure fluctuations and rapid fluid shifts following several therapies for NMO seemed to occur in AQP4 seropositive patients with PRES [28]. DWIs and ADC maps can differentiate vasogenic edema from cytotoxic edema. And the former always produces hyperintensity on ADC [33]. In our study, DWIs and ADC maps were done at acute relapse in nine patients with EBLs, and 100% of the patients demonstrated increased ADC values, which showed the occurrence of vasogenic edema. However, all of them showed hyperintense on DWIs, probably the result of T2 shine-through [7]. The brain lesions adjacent to the ventricular system mirror the periventricular and hypothalamic localization of AQP4 [6]. The extensive hemispheric lesions differed from those observed in regions of high AQP4 expression, which might be related to vasogenic edema. Based on the above, it seems reasonable to assume that EBLs in patients with anti-AQP4 antibody consist of profuse vasogenic edema associated with acute inflammation to a great extent.

### Serum complement levels in NMO with EBLs

Our previous study had shown the serum levels of C4 and CH50 were significantly higher in NMO patients than those in MS patients [34]. However, few studies focused on the changes of complement in NMO with EBLs. In the present study, serum levels of C3 and C4 in EBLs were significantly higher than those in NEBLs and NEBLs*. This observation is similar to the report of Doi et al., who found the frequency of hypercomplementemia was significantly greater in patients with extensive CNS lesions [35]. In Sjögren’s syndrome and SLE, hypocomplementemia is especially related to acute exacerbation [14,15], reflecting complement activation and consumption in vivo. AQP4-seropositive NMO is frequently associated with these systemic autoimmune diseases, such as Sjögren’s syndrome and SLE [36]. Anti-AQP4 antibody is pathogenic in NMO, which is also the main component of the IgG1 subclass. The NMO lesions are initiated by AQP4-Ab binding AQP4 and activating human complement through the classical pathway, which destroys astrocyte foot processes [12,13]. And the inflammation and demyelination, the characteristics of NMO, are secondary to classical complement activation.

### Serum CRP and ESR levels in NMO with EBLs

ESR is affected by a multitude of compounding factors, such as age, anemia and hypoalbuminemia; whereas CRP is affected only by the presence or degree of inflammation [20]. In the present study, none of the patients had systemic complications (such as upper respiratory tract infections, pneumonia or lower urinary tract infections) and majority of the patients were young adults. The values of ESR and CRP were affected to a small degree by those compounding factors. It was observed that, both the serum CRP/ESR levels and frequencies of high CRP/ESR in EBLs were higher than those in NEBLs and NEBLs* when the lesions emerged. This occurrence is associated with systemic inflammatory reactions as shown by high CRP/ESR values, which is similar with the report of Doi et al., who also found the frequency of high CRP levels (> 0.1 mg/L) was significantly higher in NMO at relapse [35]. We infer that NMO with EBLs may have a more severe inflammatory response and the occurrence of EBLs can indicate a higher disease inflammatory activity to some extent compared to NMO patients without EBLs. The CRP and ESR values obtained during the 2-years’ follow-up in EBLs and NEBLs showed that CRP decreased rapidly after the emergence of EBLs or clinical symptoms relating to EBLs. However, ESR changed slowly. Of importance, the plasma level of CRP is affected almost entirely by the synthesis rate, thus concentrations of CRP in the plasma directly reflect the degree and extent of tissue injury or inflammation. CRP levels decrease quickly once the stimulus for synthesis ceases. These physiologic properties suggest CRP is extremely useful for monitoring disease activity.

An association between high CRP/ESR and the occurrence of EBLs may indicate a role of such systemic inflammatory reactions in the formation of lesions. It is interesting to note that there are no differences in the levels of CRP and ESR between AQP4-seropositive NMO patients and AQP4-seronegative NMO patients (Tables 4 and 5) after excluding the influence of EBLs in our study. This result suggests that systemic inflammatory reactions tend to follow the presence of EBLs, regardless of the status of anti-AQP4 antibody. Initiation of inflammation at presentation of EBLs may occur independently from the existence of anti-AQP4 antibody in these conditions. These findings support the inflammation has an effect on the forming of EBLs in NMO and suggest that systemic inflammation may be a key component in the forming of EBLs.

**Table 5:** Comparison of Serum Analysis between AQP4 (−) patients and AQP4 (+) patients

**Variable**	**AQP4(−) patients (n=16)**	**AQP4(+) patients (n=53)**	**P values**
Serum analysis			
CRP (mg/l)	1.81 ± 0.65	8.78 ± 3.30	0.253
ESR (mm/H)	17.19 ± 8.86	20.91 ± 20.91	0.493
C3 (g/l)	1.16 ± 0.20	1.24 ± 0.40	0.437
C4 (g/l)	0.29 ± 0.16	0.26 ± 0.18	0.326
CH50 (U/ml)	49.50 ± 10.30	46.13 ± 11.45	0.296

*AQP4 (−)* anti-aquaporin-4 antibody negativem, *AQP4 (+)* anti-aquaporin-4 antibody positive;

*AQP4* aquaporin-4.

In our study, we also found each of serum CRP and ESR level had a positive correlation with EDSS scores respectively. Elevated serum CRP and ESR levels were associated with a poorer disability in NMO with EBLs, rendering CRP and ESR as potential surrogate markers of subclinical inflammatory activity in NMO with EBLs. The results are likely to display an aspect of anti-inflammatory treatment in NMO with EBLs and emphasize early initiation of disease modifying, anti-inflammatory therapy of NMO with EBLs.

## Conclusions

In conclusion, our findings for the first time analyzed the clinical symptoms of brain involvement and MRI characteristics of NMO with EBLs and found the changes of inflammation markers (CRP and ESR) and complement (including C3, C4, and CH50) took place obviously in the NMO with EBLs compared to the NMO patients without EBLs. These results suggest that systemic inflammation is a key component in the forming of EBLs in NMO patients. CRP is a useful marker for monitoring disease activity, which emphasizes early initiation of disease modifying and anti-inflammatory therapy in NMO with EBLs.

## Competing interests

None of the authors have any sources of support or conflicts of interests in regards to this article.

## Authors’ contributions

Study concept and design: YJ, CC, XC and XH. Acquisition of data: CC, YJ, and ZK, YD. Analysis and interpretation of data: CC, YJ, XC, YD and FP. Drafting of the manuscript: YJ and CC. Critical revision of the manuscript for important intellectual content: XC, XH, FP, ZL, CC and YJ. Statistical analysis: CC and YJ. Administrative, technical, and material support: CC, ZK, and YJ. Study supervision: XH, ZL, XC, YJ and CC. All authors red and approved the final manuscript.

## Pre-publication history

The pre-publication history for this paper can be accessed here:

http://www.biomedcentral.com/1471-2377/13/72/prepub
